# Supplementary material for: Identification and functional analysis of non-coding regulatory small RNA FenSr3 in Bacillus amyloliquefaciens LPB-18
Source: PeerJ. 2023 May 15;11:e15236. doi: 10.7717/peerj.15236 (PMC10194069; doi:10.7717/peerj.15236)
Supplement: Supplemental Information 4 [file peerj-11-15236-s004.zip › KO/CK-vs-T1_map/map04917.html]

KEGG PATHWAY: Prolactin signaling pathway - Reference pathway


|  |  |
| --- | --- |
| **Prolactin signaling pathway - Reference pathway** |  |

[
Pathway menu
| Organism menu
| Pathway entry
| Show description
| User data mapping
]

Prolactin (PRL) is a polypeptide hormone known to be involved in a wide range of biological functions including osmoregulation, lactation, reproduction, growth and development, endocrinology and metabolism, brain and behavior, and immunomodulation. PRL mediates its action through PRLR, a transmembrane protein of the hematopoietin cytokine receptor superfamily. At the protein level, the long PRLR isoform (long-R) and several short PRLR isoforms (short-R) have been detected. Acting through the long-R, PRL activates many signaling cascades including Jak2/Stat, the major cascade, Src kinase, phosphatidylinositol-3-kinase (PI3K)/AKT, and mitogen-activated protein kinase (MAPK) pathways. PRL cannot activate Jak2/Stat5 through the short-R, but can activate pathways including MAPK and PI3K pathways.

|  |  |  |
| --- | --- | --- |
| Reference pathway | 184% 150% 122% 100% 82% 67% 55% | 图片下载 |
